# pyHiM: a new open-source, multi-platform software package for spatial genomics based on multiplexed DNA-FISH imaging

**DOI:** 10.1186/s13059-024-03178-x

**Published:** 2024-02-13

**Authors:** Xavier Devos, Jean-Bernard Fiche, Marion Bardou, Olivier Messina, Christophe Houbron, Julian Gurgo, Marie Schaeffer, Markus Götz, Thomas Walter, Florian Mueller, Marcelo Nollmann

**Affiliations:** 1 Centre de Biologie Structurale, Univ Montpellier, CNRS UMR 5048, INSERM U1054, 34090 Montpellier, France; 2 Centre for Computational Biology (CBIO), Mines Paris, PSL University, 75006 Paris, France; 3 Institut Curie, 75248 Paris, Cedex France; 4 INSERM, U900, 75248 Paris, Cedex France; 5 Imaging and Modeling Unit, Institut Pasteur, Université Paris Cité, Paris, France

**Keywords:** Spatial genomics, 3D chromatin structure, Transcription, Imaging, Bioimage informatics

## Abstract

Genome-wide ensemble sequencing methods improved our understanding of chromatin organization in eukaryotes but lack the ability to capture single-cell heterogeneity and spatial organization. To overcome these limitations, new imaging-based methods have emerged, giving rise to the field of spatial genomics. Here, we present pyHiM, a user-friendly python toolbox specifically designed for the analysis of multiplexed DNA-FISH data and the reconstruction of chromatin traces in individual cells. pyHiM employs a modular architecture, allowing independent execution of analysis steps and customization according to sample specificity and computing resources. pyHiM aims to facilitate the democratization and standardization of spatial genomics analysis.

**Supplementary Information:**

The online version contains supplementary material available at 10.1186/s13059-024-03178-x.

## Background

In eukaryotes, the three-dimensional (3D) nuclear organization of chromatin is tightly controlled and plays an active role in gene regulation, DNA replication and DNA damage repair. In the last decade, genome-wide ensemble methods, such as Hi-C and 3C [1], have revolutionized our understanding of genome structure at the megabase-to-kilobase scale by revealing the complex organization of chromatin into compartments, topologically associating domains, and chromatin loops [2, 3]. However, these bulk approaches are unable to dissect single-cell heterogeneity or preserve spatial information in tissue [4–6].

**Fig. 1:**
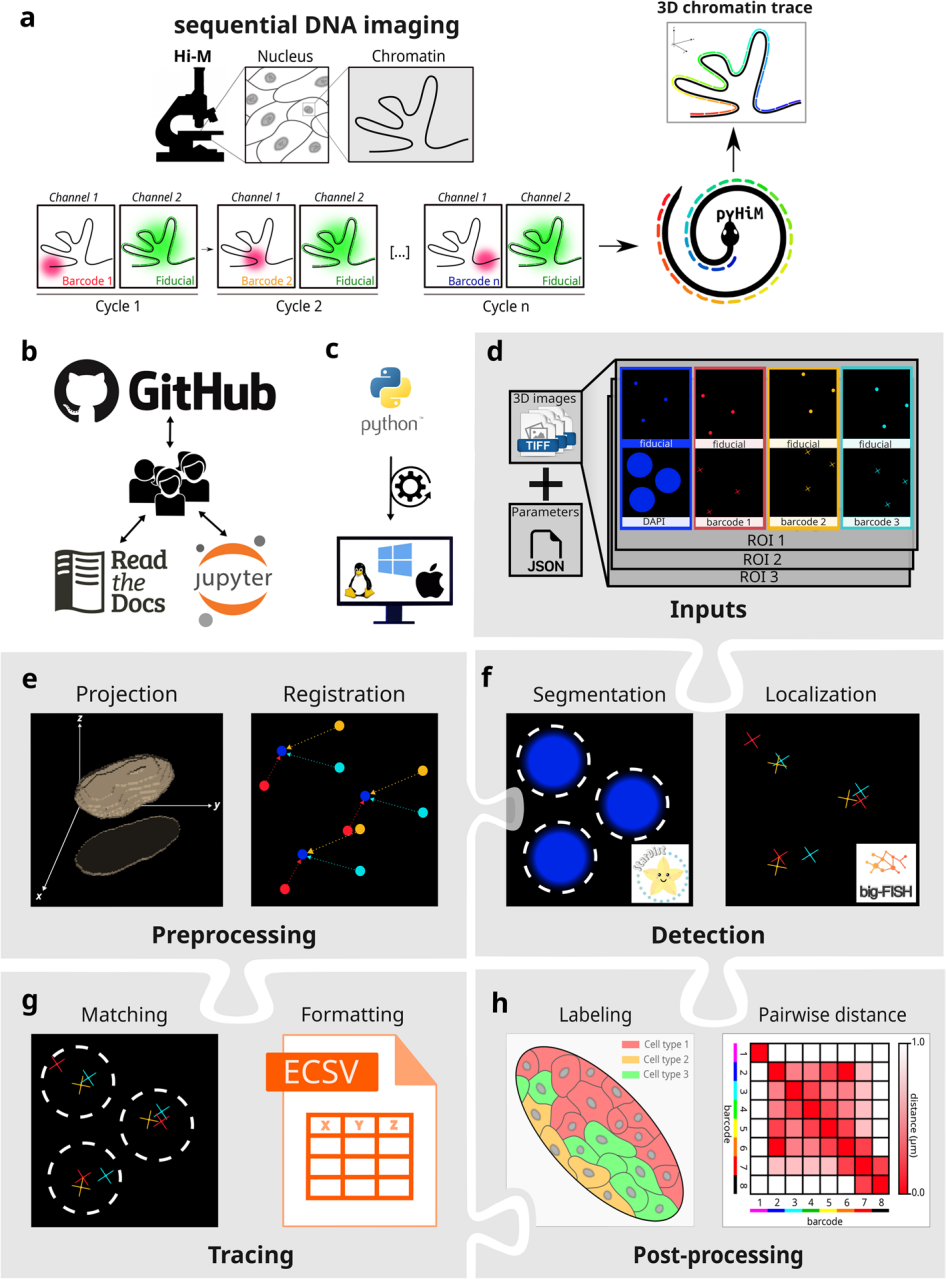
**a** Schematic description of Hi-M microscopy: Chromatin is imaged through multiple acquisition cycles, each targeting a specific genomic locus using a set of unique DNA-FISH oligonucleotides targeted by a complementary, fluorescently labeled oligonucleotide. A fiducial marker is simultaneously imaged to allow for registration and drift correction during post-processing. Using pyHiM, the 3D conformation of the target locus is reconstructed for each individual cell. **b** pyHiM is an open-source project hosted on GitHub. Extensive documentation and Jupyter notebooks are available for users and developers. **c** pyHiM is developed in Python and runs indifferently on Linux, Windows and macOS. **d** Input data: 3D images are organized by imaging channel (DAPI, fiducial, DNA-FISH spots, etc.) and FOV. A single json file combines all parameters needed to run the analysis pipeline. **e** 3D images are pre-processed by calculating the maximum intensity projection and applying 2D registration based on the fiducial images. **f** Masks for nuclei, oligopaint libraries, and DNA-FISH spots are computed using pre-trained deep learning models. Individual DNA-FISH spots are localized with sub-pixel accuracy using apiFISH (fork of big-FISH). **g** Individual traces are built by combining the localizations of all DNA-FISH spots detected within the same mask. Results are saved in ECSV format. **f** Post-processing analyses are performed to obtain pairwise distance and proximity frequency matrices for each combination of DNA loci and for different spatial regions of the sample containing different cell types

Recently, a new family of imaging-based methods was developed to trace the 3D conformation of chromatin in single cells, giving rise to the field of spatial genomics [7–11] (Fig. 1a). These techniques perform sequential imaging of genomic loci with a precision of a few tens of nanometers, allowing for the 3D mapping of a given region of chromatin at kilobase resolution in thousands of individual cells [8, 9, 12]. Our specific implementation, called Hi-M, couples detection of chromatin structure and transcriptional output [8] (Fig. 1a). Since their creation, spatial genomics methods based on sequential imaging were successfully used for the detection of short- and long-range chromatin interactions in multiple model systems, including mammalian cultured cells, fly embryos, and mouse tissues [7–10, 12]. Critically, imaging-based spatial genomics technologies complement transcriptomic surveys of single cells in their spatial context and thus have the potential to lead to important new discoveries in multiple fields, including 3D genomics, transcriptional regulation, DNA replication, or DNA repair.

In recent years, several efforts were made to promote a wider use of these new technologies by sharing experimental and image analysis protocols [7, 13–16]. However, democratization of spatial genomics will require the development of open-source and user-friendly software packages for reconstructing chromatin traces (i.e., unique sets of 3D coordinates describing a locus conformation in an individual cell) from raw, 3D, multicolor images [17]. To this end, such software should (1) provide access to validated cutting-edge techniques required for the analysis of spatial genomics data, (2) use a license-free programming language, (3) provide extensive documentation and tutorials to guide new users and allow development of new functionalities, (4) adopt a modular architecture to facilitate adaptation to future developments in spatial genomics, and (5) use novel analysis methods to ensure robust, automatic analysis of large data sets (several Tb per experiment) without user input and in reasonable times.

## Results

To address these needs, we introduce pyHiM, an open-source, modular and scalable software toolbox specifically designed for sequential spatial genomics data analysis (Fig. 1a). pyHiM comes with extensive user and developer documentation, Jupyter Notebook tutorials, and a DNA-FISH dataset to guide new users through the main steps of a typical analysis pipeline (Fig. 1b). PyHiM can be easily installed using standard package management tools (conda and PyPi, Additional file 1: Fig. S1a) and conveniently runs on Linux, Windows, and macOS (Fig. 1c). A single human-readable configuration file is used to centralize all analysis parameters and can be edited thanks to a user-friendly graphical user interface (GUI) (Additional file 1: Fig. S1b). In addition, a command-line interface enables execution on multiple hardware platforms, from laptop computers to high-performance computing (HPC) clusters. Functionality can be tuned according to local hardware specifications, acquisition conditions (e.g., number of channels, size of 3D image stacks), and sample properties.

The analysis pipeline of pyHiM is organized in modules, each performing a specific analysis task. The inputs of pyHiM are 3D image stacks in the universal TIFF format (Fig. 1a, d). Deconvolution of images before pyHiM execution is not mandatory for pyHiM analysis but, in our experience, improves the quality of the results and the statistics of reconstructed chromatin traces.

The pre-processing module organizes images by field of view (FOV) and by the type of probe imaged: DNA-FISH spots, nuclear/ oligopaint library masks, fiducial marks, or RNA expression. For each FOV, pyHiM first performs a projection and global registration using fiducial images acquired at each cycle as references (Fig. 1e). To improve the robustness of this step, we implemented a new method whereby the image is decomposed in blocks that are independently co-aligned. A polling step then determines the most popular global registration and applies it to the whole image (Additional file 1: Fig. S2a). This step allows for a global correction of thermal drift and stage repeatability error even for cycles with fiducial images displaying local distortions. Samples such as embryos or tissues may often display local deformations during acquisition of different cycles which cannot be taken into account by global registration algorithms. Thus, we developed a new local registration algorithm that optimizes 3D registrations locally to correct for 3D sample deformations (Additional file 1: Fig. S2b, c).

The spot detection module performs segmentation and localization of DNA-FISH spots with sub-pixel accuracy of all sequential imaging rounds, using a combination of Deep Learning (DL)-powered spot segmentation followed by robust and automated 3D Gaussian fitting. 3D-DL segmentation is performed using a StarDist neural network [18] trained to robustly detect 3D-Point Spread Functions (PSF) in diverse sample types and illumination conditions. We obtained this network after extensive simulations of PSFs with different signal-to-noise ratios and inhomogeneous background levels. Next, based on the centroid position of each DL-mask, a robust 3D Gaussian fit of the intensity distribution is performed using apiFISH (Fig. 1f and Additional file 1: Fig. S3) [19].

The mask detection module segments nuclei in 3D using pre-trained StarDist neural networks models [18] (Fig. 1f). Other custom models based on StarDist or other popular architectures (e.g., Cellpose [20]) can also be integrated via a plugin. Finally, DNA-FISH spots localized within the same mask are combined into chromatin traces, which are assigned a universally unique identifier and tabulated in human-readable Enhanced Character-Separated Values (ECSV) format (Fig. 1g and Additional file 1: Fig. S4a, b). Additional labels, based on RNA expression levels or spatial cell distribution, can be assigned to each single trace, allowing for cell/tissue-specific post-processing analysis (Fig. 1h).

**Fig. 2:**
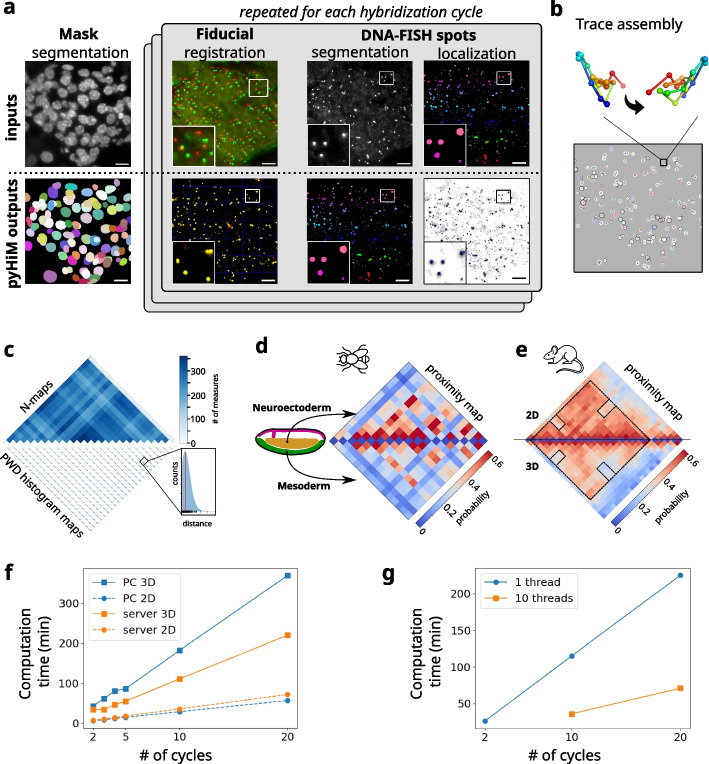
**a** Illustration of a typical pyHiM analysis on mouse tissues: examples of raw data are shown in the top row and the most relevant pyHiM outputs are shown in the bottom row. From left to right, raw DAPI data are segmented to compute the 3D masks of each individual nucleus. Next, 2D and 3D registration of the fiducial is performed for each imaging cycle, and the quality of the correction can be quickly assessed based on the output image. Then, the localization of individual DNA-FISH spots is performed in two steps: first, a 3D mask of each DNA-FISH spot is computed using deep learning. Then, using the mask position as a reference, the sub-pixel localization of the spot is inferred using apiFISH. Scale bars = 8 μm. **b** Chromatin tracks are calculated by combining all individual DNA-FISH spot localizations detected within the same mask (DAPI, or locus). Each individual trace represents a snapshot of the locus conformation within a single cell (see reconstruction with two different orientations). **c** Data quality assessment: (top) the N-matrix represents the number of times that each pair of DNA loci was detected in the dataset and is indicative of their detection efficiency. (bottom) The distribution of pairwise distances between DNA-FISH spots in the same chromatin trace is plotted to ensure that there is no major error in the analysis (detection threshold, etc.). **d** Traces computed by pyHiM were sorted based on RNA expression profiles in NC14 fly embryos and assigned to specific cell types (e.g., mesoderm vs. neuroectoderm). Specific long-range interactions and chromatin organization are observed for each cell type. **e** Fast 2D analysis based only on the projected 3D data is used to optimize parameters and test data quality. An example from mouse tissue data shows the pairwise distance maps computed using 2D (top) and 3D (bottom) analysis. The 2D map captures most of the features that characterize the conformation of the locus. **f** Comparison of pyHiM execution times for different number of cycles and for a desktop computer (Intel(R) Core(TM) i7-8700 CPU @ 3.20 GHz, CPUs: 12, cores: 6, threads per core: 2, memory: 16 Gb) or a multi-threaded server (AMD EPYC 7702 64-Core Processor 3.34 GHz, CPUs: 256, cores: 128, threads per core: 2, memory: 512 Gb). **g** Performance of pyHiM using single-threaded or DASK-powered multi-threading

Thanks to pyHiM’s modular architecture, each analysis step in the pipeline (registration, detection, tracing, etc.) can be run independently. Users can tailor the analysis workflow according to their sample specificity, acquisition conditions, and available computing resources (Fig. 2a). Intermediate results, such as unfiltered localizations or traces, are saved in ECSV format after each module execution, allowing the user to perform custom data validation or additional analysis. Finally, each module produces reports in human-readable markdown files with snapshot images illustrating the performance of the analysis for each cycle and FOV. This allows the user to efficiently assess the quality of the analyses and eventually fine-tune parameters to improve them (Fig. 2a–c and Additional file 1: Figs. S2, S3). pyHiM can successfully analyze experimental data acquired from a variety of sample types, ranging from fly embryos to mouse and human tissues (Fig. 2d, e).

pyHiM also offers a number of additional features that facilitate data formatting, result display, and post-processing. For instance, DNA-FISH spot detection efficiency and maps of the pairwise distance (PWD) distributions between DNA-FISH spots from different cycles (Fig. 2c), or proximity frequency matrices for specific cell types (Fig. 2d). Another important feature of pyHiM is its ability to perform rapid analysis in 2D (Fig. 2e). In this mode, pyHiM projects signals from DNA-FISH spots and masks in 2D and performs registration, segmentation, spot localization, and tracing in 2D. Contact maps computed using the 2D pipeline show all the relevant features of 3D maps (long-range contacts, TADs, etc.) but require ~ 5 × less computation time (Fig. 2e, f) and can therefore be used to quickly assess the quality of the acquired dataset before full 3D analysis.

Finally, a critical aspect of multiplexed DNA-FISH imaging is the amount of data generated, typically ~ 1–3 Tb per experiment depending on the number of cycles and the number of FOVs. To handle and analyze such large volumes of data in a reasonable time, we have implemented a parallelization mode based on the Dask Python package. For this, pyHiM analyzes data associated with different hybridization cycles in parallel, while keeping the technical aspects transparent to the user, leading to a drastic shortening in computation time (Fig. 2f, g). Conveniently, a reporting web-server based on Bokeh can be launched to monitor analysis status and performance in real-time (Additional file 1: Fig. S4c). As a result, pyHiM can run indifferently on a laptop or an HPC cluster and be tuned according to the technical specificities of both (e.g., number of CPUs, available memory, availability of GPUs, etc.).

## Conclusions

In summary, we describe pyHiM, a modular, user-friendly, well-documented tool for chromatin tracing analysis based on sequential DNA-FISH imaging. pyHiM can be used to analyze data produced by Hi-M or by other spatial genomics methods. Thus, we envision that the adoption of pyHiM will enable the growth of a new user community for this active field of research. Indeed, as data acquisition and sample preparation become standard and even commercially available, a final bottleneck for widespread adoption will be the availability of flexible image analysis tool boxes dedicated to chromatin tracing. Thus, a well-tested and user-friendly analysis pipeline such as pyHiM will be key to break barriers to adoption of spatial genomics by users and microscopy facilities, to promote transparent image analysis pipelines in the field, and to create a large user community to accelerate discoveries and new developments. The modularity, open-source nature, and extensive developer documentation of pyHiM were purposefully designed to promote collaborative developments, to standardize and benchmark image analysis practices, and to facilitate reuse of existing algorithms to implement analysis tools for novel technologies in the blooming field of spatial genomics.

## Methods

### Inputs

The two minimal inputs of pyHiM are as follows: a dictionary of parameters (*parameters.json*) and a list of images to process. *parameters.json* contains acquisition parameters (e.g., pixel size), file formatting parameters (e.g., regular expression to decode filenames), and all the parameters that are required for the execution of each module in pyHiM. For detailed information on the *parameters.json* parameter file, please refer to our online resource: Input Parameters.

Input images can be of two types: DNA-FISH spots for a given cycle and masks used for tracing. The latter can be either nuclear masks (e.g., from DAPI labeling) or from a cycle where the whole oligopaint library is labeled and imaged at once. Both DNA-FISH and mask images must be accompanied by a corresponding fiducial image used for registration (see the “Registration” section). Images are assumed to be in the universal and non-proprietary TIFF format. Use of deconvolved images is recommended but not compulsory.

### Projection

We developed a tool for image reprojection (module: *makeProjections*). This step is necessary for lateral global drift alignment (see the”Registration” section) and for the rapid visual inspection of input files. Sum and maximum projections are implemented and configured through the *parameters.json* parameters file. We recommend the former for masks and the latter for DNA-FISH images. makeProjections allows for the manual selection of the *z*-range and implements an automatic algorithm to robustly retrieve the in-focus plane. Briefly, this method estimates the optimal in-focus plane by calculating the maximum of the Laplacian of the intensity profile along the *z*-axis. The calculation is performed block-by-block to take into account local variability and sample drift. More details on the methods and the execution of this module can be found in the online description of the *makeProjections* module.

### Registration

We implemented two registration methods to obtain automatic and robust global and local realignments. The *alignImages* module performs global realignments by registering the 2D z-reprojected fiducial images using 2D cross-correlation. This method, however, can be unreliable when fiducial images contain impurities that vary between cycles. To solve this, we developed a second algorithm (*alignByBlock*) that uses block-by-block decomposition to determine the best registration for each block. This calculation is followed by a polling operation that retrieves the most satisfactory global registration. This second method is highly robust to impurities. More details on the methods and the execution of this module can be found in our online description of the *alignImages* module. Once registrations for each cycle are processed, the module *appliesRegistrations* re-interpolates 2D images of DNA-FISH spots and masks to provide a visual input of the performance of global registrations for each hybridization cycle.

Biological samples can display local deformations (typically in the hundreds of nm range) during the long-term acquisition times of a HiM dataset. These distortions cannot be properly corrected by global 2D realignment routines. To tackle this issue, we developed a new registration method that performs local 3D registration. In this method, images are first globally realigned in 2D. Next, fiducial images are decomposed in 3D blocks and each block is realigned by 3D cross-correlation and re-interpolation. The resulting local block corrections are stored as an ASTROPY table [21] that is used by the *register_localizations* module (see section below). More details on the methods and the execution of this module can be found in our online description of the *alignImages3D* module.

### Segmentation and detection

Three different modules were built to deal with the segmentation and detection of DNA-FISH spots and masks. First, we developed a module for the segmentation and localization of masks and sources in 2D (module: *SegmentMasks*). Mask and DNA-FISH images are segmented using startdist with pre-trained networks. Segmented objects are filtered by size and shape, while merged objects are split using the watershed algorithm. DNA-FISH spots are fitted using the highly efficient DAOStarFinder algorithm from photutils [22] and post-processed using *filter_localizations*.

Second, we developed a module specifically designed to segment masks in 3D (module: *segmentMasks3D*). *segmentMasks3D* relies on deep-learning segmentation using a network that we trained specifically to robustly segment nuclei in 3D with stardist [23]*.* Other DL segmentation tools, such as cellpose [20], can be used to further increase the flexibility of mask segmentation for different biological samples (script: mask_cellpose.py). *segmentMasks3D* then post-processes 3D masks by size and shape filtering, and applies a watershed algorithm to split merged masks. The output of *segmentMasks3D* is a localizations table used by the *build_traces* module to group localizations into single chromatin traces (see the “Tracing” section) (Additional file 1: Fig. S3c). More details on the methods and the execution of this module can be found in our online description of the *segmentMasks3D* module.

Finally, we developed a module for the segmentation and localization of DNA-FISH spots (module: *segmentSources3D*). *segmentSources3D* segments DNA-FISH spots by using a stardist DL network trained to detect PSFs in 3D. This network was optimized by training the DL network on simulated data displaying large variations in signal-to-noise ratios, local background inhomogeneities, and intensity levels. After segmentation, *segmentSources3D* fits the intensity distributions within DNA-FISH spot masks with a 3D-Gaussian model using non-linear regression with functions from apiFISH [19]. The output *segmentSources3D* is an ASTROPY table containing the *xyz* coordinates, identities, and properties of all the localizations. Localizations with low intensities are filtered in post-processing using the module *filter_localizations*. A final step before tracing involves the application of local registrations to the localization tables obtained from *segmentMasks* or from *segmentSources3D using the register_localizations module.* Our trained stardist DL networks were packed with pyHiM and are also available from our pyHiM OSF repository.

### Tracing

The final step involves the grouping of DNA-FISH spots belonging to the same chromatin fiber (module: *build_traces*). This can be accomplished in two manners. The first involves spatial clustering based on nearest-neighbor distances with the KDTree algorithm. This method works well for low-density samples, where nuclei are well-separated in space. The second, instead, relies on the use of user-provided masks. In this case, traces are built by grouping together the spots belonging to the same mask. Masks can be derived either from nuclei (e.g., from DAPI staining) or from a cycle labeling the entire oligopaint library. The output of *build_traces* is a trace table in ASTROPY format where each trace is stamped with a universal unique identifier to enable the automatic merging of multiple trace tables and to ensure traceability. We note that no corrections for missing or redundant barcode localizations are applied by *build_traces* as such corrections are often sample dependent. In pyHiM, these issues are handled by post-processing scripts (see below). More details on the methods and the execution of this module can be found in our online description of the *build_traces* module.

We developed several tools for post-processing of trace tables. *Trace_assign_mask* finds traces that match specific morphological or gene-expression patterns by matching trace localization with user-provided masks. *Trace_combinator* and *trace_merge* fuse traces from different FOVs or different experiments. *Trace_filter* is a general tool for filtering traces that can remove specific barcodes from a trace table, remove duplicated localizations from single traces, and perform spatial filtering. *Trace_filter_advanced*, instead, removes duplicate localizations based on distance constraints. Finally, *trace_analyzer* analyzes a trace table to calculate the distribution in the number of barcodes detected per trace, the number of times each barcode appears in single traces, and the spatial clustering of traces. We highlight the existence of more sophisticated methods to improve the quality of tracing [24] that may be applied in combination with the post-processing scripts mentioned in this paragraph.

Finally, we developed an algorithm that builds maps from trace tables (module: *build_matrix*). This tool produces conventional pair-wise median distance maps by relying on kernel-density estimators to accurately calculate the maximum of each distance distribution, and calculates proximity distance maps for user-specified threshold distances. *Build_matrix* produces N-maps which contain the number of localizations detected for each combination of barcodes, a diagnostic tool that is fundamental to determine the performance of an experiment and the robustness of detection for each barcode pair. More details on the methods and execution of this module can be found in our online description of the *build_matrix* module.

### Supplementary Information

**Additional file 1.** Supplementary figures.

**Additional file 2.** Review history.

## Data Availability

The latest stable and development versions of pyHiM are publicly available at our GitHub repository: https://github.com/marcnol/pyHiM [25]. The 0.9.1 release of pyHiM is permanently available at: https://osf.io/updfw (10.17605/OSF.IO/UPDFW). The online documentation is available at https://pyhim.readthedocs.io/en/latest/ [26]. DL networks trained for pyHiM are available at our OSF repository: https://osf.io/ugpyh/ [27]. Minimal datasets for multiplexed DNA-FISH data are available from https://osf.io/6egdc/ [28].
