# Supplementary material for: pyHiM: a new open-source, multi-platform software package for spatial genomics based on multiplexed DNA-FISH imaging
Source: Genome Biol. 2024 Feb 13;25:47. doi: 10.1186/s13059-024-03178-x (PMC10863255; doi:10.1186/s13059-024-03178-x)
Supplement: Supplementary file 1 — Additional file 1. Supplementary figures. [file 13059_2024_3178_MOESM1_ESM.pdf]

Supplementary Figures for

pyHiM: a new open-source, multi-platform software package for spatial genomics based on multiplexed DNA-FISH imaging

Devos Xavier, Fiche Jean-Bernard, Bardou Marion, Messina Olivier, Houbron Christophe, Gurgo Julian, Schaeffer Marie, Götz Markus, Thomas Walter, Florian Mueller, Nollmann Marcelo

Fig. S1 2
Fig. S2 3
Fig. S3 5
Fig. S4 6

Fig. S1

a

pyHiM dependencies

Library	Version
apifish	>= 0.6.4
astropy	>= 4.3.1
csbdeep	>= 0.6.3
dataclasses-json	>= 0.6.0
dask[distributed]	>= 2023.4.1
matplotlib	== 3.5.1
numba	>= 0.54.1
numpy	>= 1.19.5
photutils	>= 1.1.0
pylab	>= 1.3.2
pympler	>= 1.0.1
roipoly	>= 0.5.3
scipy	>= 1.7.3
scikit-image	== 0.19.2
scikit-learn	>= 1.1.1
stardist	>= 0.7.3
tifffile	>= 2021.10.10
tqdm	>= 4.63.0

b

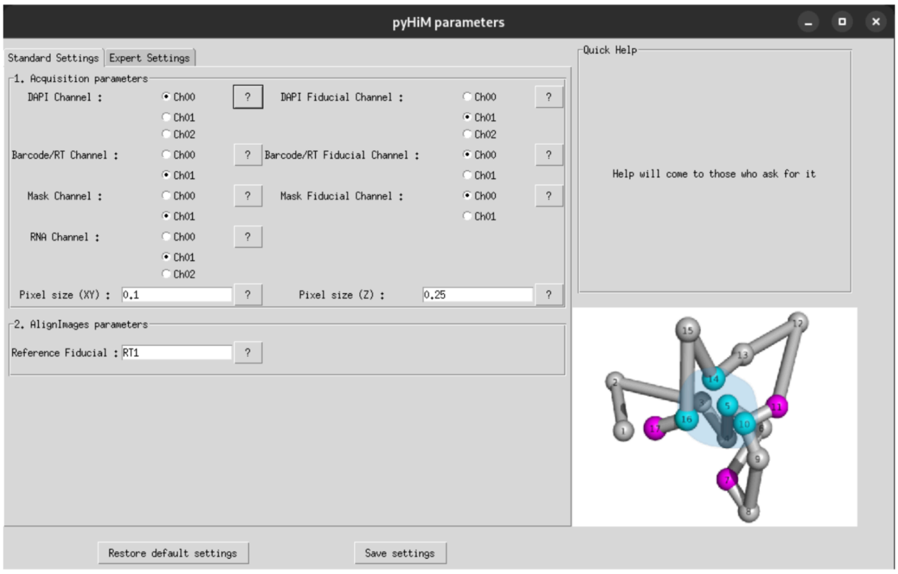

Table S1a List of all the python Packages used by pyHiM and their versions.
Fig. S1b Graphical user interface for pyHiM parameter setting routine.

Fig. S2

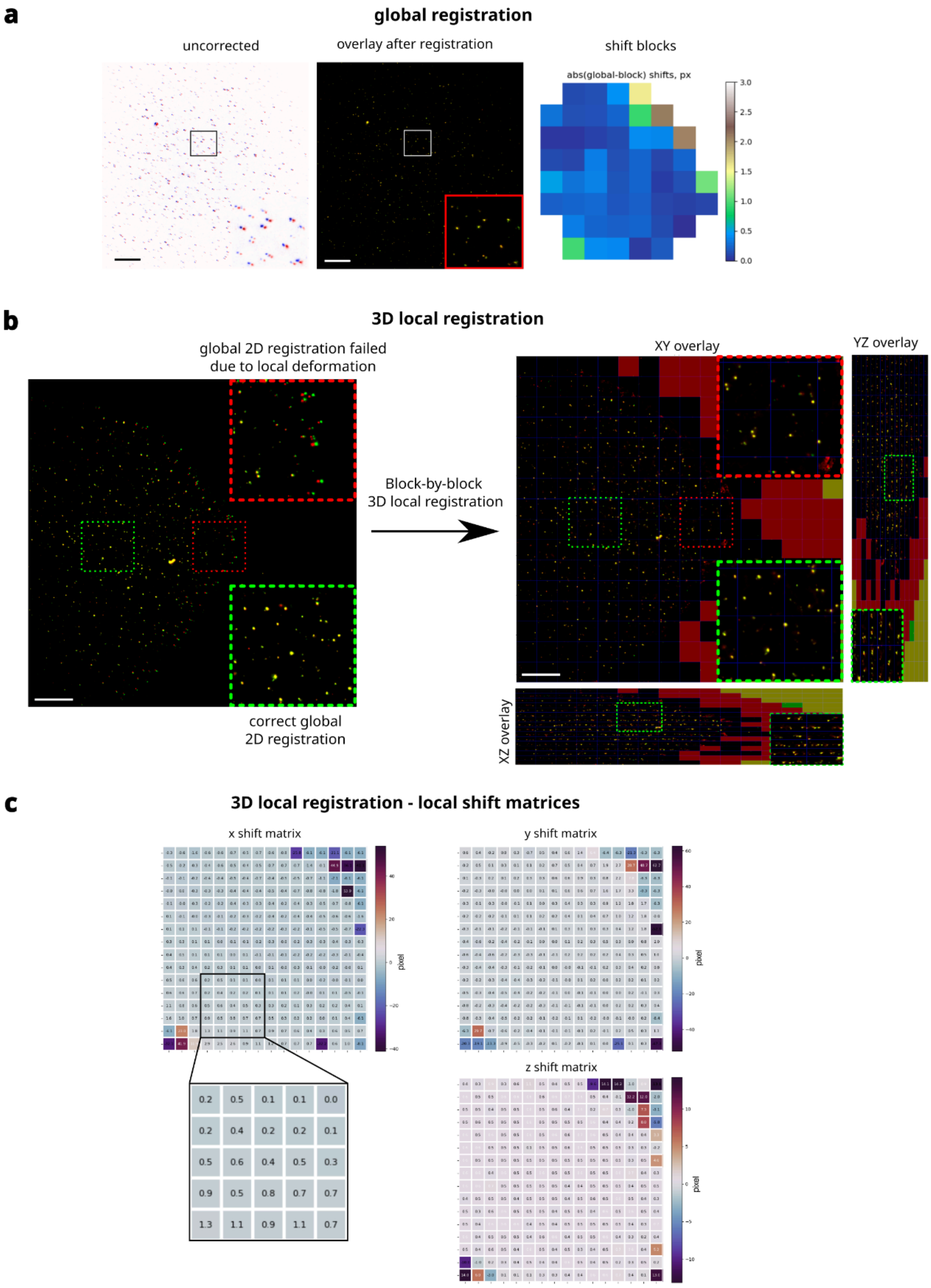

a- pyHiM first performs a 2D registration on projected 3D images: Example of two superimposed fiducial images before (left) and after (middle) registration. The registration can be performed globally or locally. In the latter case, the image is divided into an array of 8x8 blocks and the registration is computed separately for each block. The block correction map (right) shows the shift along the x-axis applied to the registered image (in pixels, 1 pixel is 105 nm). Scalebars = 25 µm.

b- The 2D global registration can be affected by local sample deformation, and a 3D block-by-block routine is used to further improve the correction. Again, a local correction is

computed by dividing the 3D images into an array of 16 x 16 x 16 blocks. A quick assessment of the correction quality can be made based on the overlaid images. Scalebars = 25 µm.
c- Average block correction maps for 3D registration are used to check for errors or unexpected deformation of the sample during the experiment.

Fig. S3

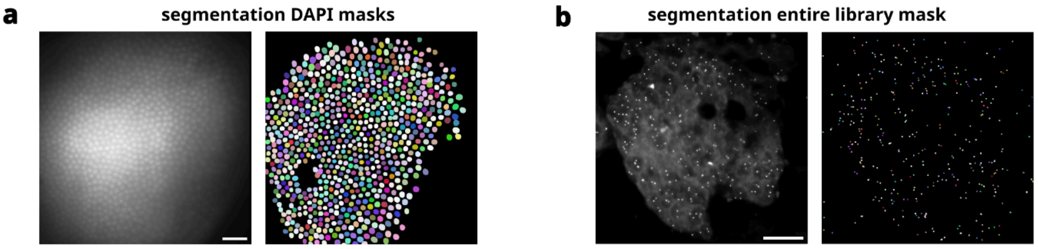

c **localizations table**

Column	Description
Buid	Unique identifier of spot
ROI #	Region of interest number
CellID #	Cell identifier number
Barcode #	DNA-FISH probe number
id	Unique spot identifier per barcode
zcentroid	Localization along z-axis (in plane)
xcentroid	Localization along x-axis (in px)
ycentroid	Localization along y-axis (in px)
sharpness	Ratio between max intensity and mean background
roundness1	Object roundness based on symmetry
roundness2	Object roundness based on Gaussian fits
npix	Number of pixels in the Gaussian kernel array
sky	Input background intensity (default is 0)
peak	Maximum pixel value of the spot
flux	Peak density divided by the detection threshold
mag	-2.5 * log10(flux)

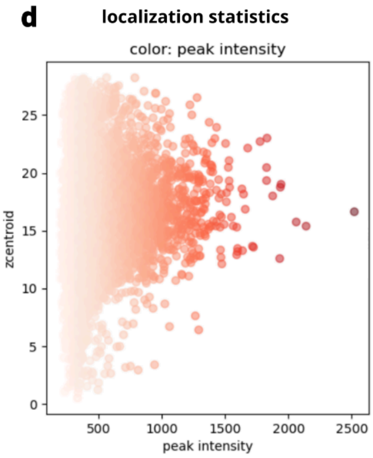

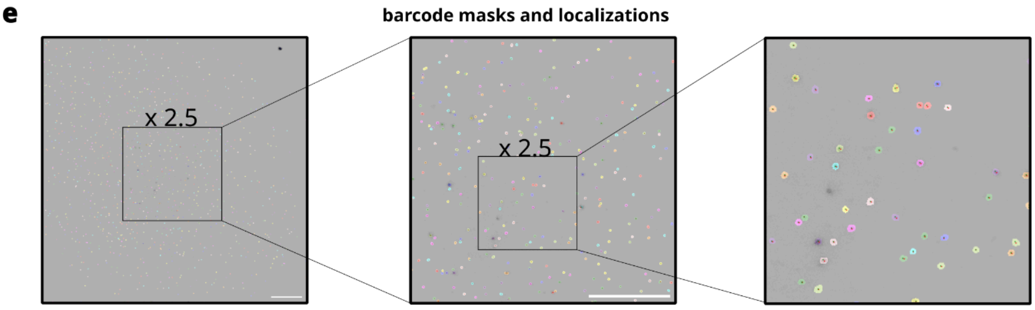

a- DAPI-stained nuclei are imaged in a Drosophila embryo (left) and segmented using StarDist (right). Scalebar = 25μm.

b- Using a library of oligonucleotides targeting a large genomic region, bright 3D fiducial images are acquired in mouse tissue (left). Using custom trained StarDist models, the images are segmented and individual masks are computed for each fiducial. Scalebar = 25μm.

c- For each round of acquisition, individual DNA-FISH images are analyzed and sub-pixel localizations are computed using big-FISH. Each detection is assigned a unique ID and saved together with its x, y, z localizations and intensity in a *.dat* file. The table summarizes all the parameters available for each detection.

d- The distribution of peak intensity is plotted against spot localizations along the z-axis. Such data can be used to further optimize the intensity threshold used for detection and check for illumination artifacts.

e- Example of detection output. Each individual detections are represented by a spot of color overlay with the maximum intensity projection of the original image (in inverted grayscale). Scalebars = 25μm.

Fig. S4

a **example traces**

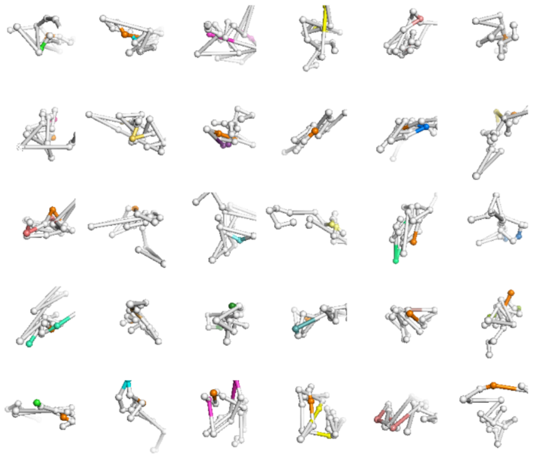

b **trace table**

Column	Description
Spot_ID	Spot unique identifier
Trace_ID	Trace unique identifier
x	x coordinate (in nm)
y	y coordinate (in nm)
z	z coordinate (in nm)
Chrom	Chromosome
Chrom_Start	Sequence start (in bp)
Chrom_End	Sequence end (in bp)
ROI #	Region of interest number
Mask_id	Mask identifier
Barcode #	DNA-FISH probe number
label	Optional label to classify trace

c **online Bokeh server**

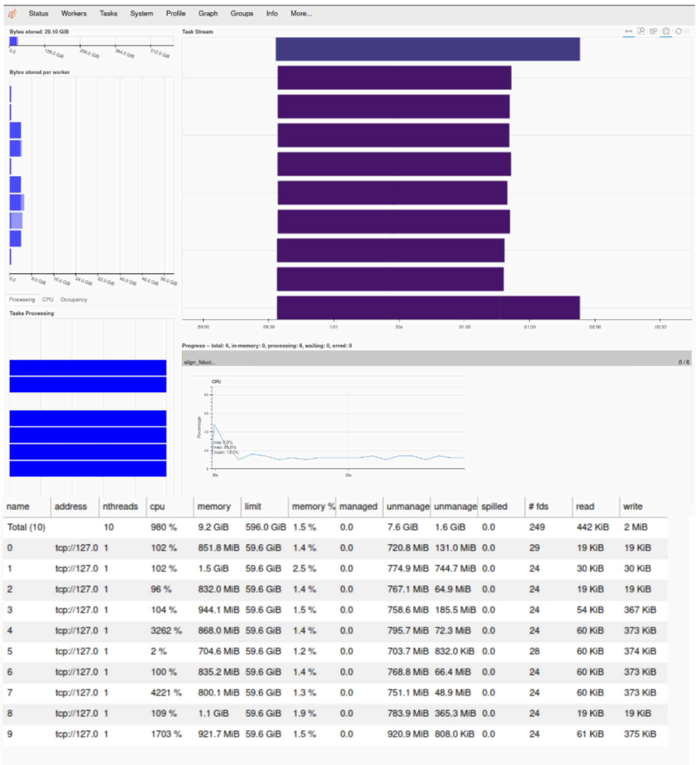

name	address	nthreads	cpu	memory	limit	memory %	managed	unmanage	unmanage	spilled	# fds	read	write
Total (10)		10	980 %	9.2 GiB	596.0 GiB	1.5 %	0.0	7.6 GiB	1.6 GiB	0.0	249	442 KiB	2 MiB
0	tcp://127.0	1	102 %	851.8 MiB	59.6 GiB	1.4 %	0.0	720.8 MiB	131.0 MiB	0.0	29	19 KiB	19 KiB
1	tcp://127.0	1	102 %	1.5 GiB	59.6 GiB	2.5 %	0.0	774.9 MiB	744.7 MiB	0.0	24	30 KiB	30 KiB
2	tcp://127.0	1	96 %	832.0 MiB	59.6 GiB	1.4 %	0.0	767.1 MiB	64.9 MiB	0.0	24	19 KiB	19 KiB
3	tcp://127.0	1	104 %	944.1 MiB	59.6 GiB	1.5 %	0.0	758.6 MiB	185.5 MiB	0.0	24	54 KiB	367 KiB
4	tcp://127.0	1	3262 %	868.0 MiB	59.6 GiB	1.4 %	0.0	795.7 MiB	72.3 MiB	0.0	24	60 KiB	373 KiB
5	tcp://127.0	1	2 %	704.6 MiB	59.6 GiB	1.2 %	0.0	703.7 MiB	832.0 KiB	0.0	28	60 KiB	374 KiB
6	tcp://127.0	1	100 %	835.2 MiB	59.6 GiB	1.4 %	0.0	768.8 MiB	66.4 MiB	0.0	24	60 KiB	373 KiB
7	tcp://127.0	1	4221 %	800.1 MiB	59.6 GiB	1.3 %	0.0	751.1 MiB	48.9 MiB	0.0	24	60 KiB	373 KiB
8	tcp://127.0	1	109 %	1.1 GiB	59.6 GiB	1.9 %	0.0	783.9 MiB	365.3 MiB	0.0	24	19 KiB	19 KiB
9	tcp://127.0	1	1703 %	921.7 MiB	59.6 GiB	1.5 %	0.0	920.9 MiB	808.0 KiB	0.0	24	61 KiB	375 KiB

a- 3D representation of example chromatin traces.

b- Format of the chromatin table output file.

c- Snapshot of Bokeh server displaying the advancement of a pyHiM execution in parallel mode.
