# Supplementary material for: pyHiM: a new open-source, multi-platform software package for spatial genomics based on multiplexed DNA-FISH imaging
Source: Genome Biol. 2024 Feb 13;25:47. doi: 10.1186/s13059-024-03178-x (PMC10863255; doi:10.1186/s13059-024-03178-x)
Supplement: Supplementary file 2 — Additional file 2. Review history. [file 13059_2024_3178_MOESM2_ESM.docx]

Review History

**First round of review**

**Reviewer 1**

**Were you able to assess all statistics in the manuscript, including the appropriateness of statistical tests used?**

Yes, and I have assessed the statistics in my report.

**Were you able to directly test the methods?**

Yes

**Comments to author:**

Xavier and colleagues describe a professionally organized tool for reconstructing 3D chromatin paths from sequential imaging data. Chromatin tracing data have proliferated substantially in recent years, but most practitioners still rebuild the wheel from scratch as it were, using custom in house software approaches for analysis due to the lack of easy to use reliable tools. This state of affairs has slowed the growth of the field and limited the opportunity for optimization (comparing and competing different approaches) that highly portable well documented software could bring. This work has significant promise to help close that gap and we are enthusiastic about seeing it published.

The modular nature of the work is an important strength. This is nicely showcased in the integration of different methodologies for cell-segmentation, including StarDist and cellpose. Rather than rebuild these from scratch, the software takes advantage of more widely used and well maintained existing software in the image processing field. Similar modularity is exhibited in the spot fitting with BigFISH and the use of existing generalist approaches for parallelization (with Dask) and performance monitoring (with Bokeh). The support for Windows/Mac/Linux environment was also commendable.

We have the following suggestions for improvement:

While refreshingly succinct, readable, and data rich, the main text could benefit (in our opinion) from a little further development in a few key areas.

We found it helpful that the authors did indeed include demo data with software online. Nonetheless, the existence of such demo data was not immediately apparent from reading the main text of the article, nor from the initial readme page of the PyHiM github repository.
The main text describes most of the key steps of the algorithm, but did not include a clear description of the spot-stitching approach. For example, how does the software handle the detection of multiple foci in the same image? Does it use the closest spot? The brightest spot? Some weighted combination of these? Completely optional: Perhaps the authors would like to add support for some recently developed (python) algorithms designed to address this issue, like the "spatial genome aligner" from the Ren lab?: https://github.com/b2jia/jie

II. Despite the elegant implementation with conda and pip, and the reasonable amount of documentation, we were ultimately unable to run even the demo data analysis "out-of-the-box". These errors no doubt reflect our own deficiencies as much as they reflect any inadequacies in the author's approach. Nonetheless, in hopes of helping improve the software and its adaptation, we summarize the problems we encountered here:

1) One problem appears to stem from some filepaths that are still hard coded:
Cannot run segmentMask3d function
FileNotFoundError: config file doesn't exist: /mnt/grey/DATA/users/marcnol/models/StarDist3D/mask_DAPI/models/stardist_20210625_deconvolved/config.json
The model in infoList_model.json still points to the developer path.
Similarly, I cannot run segmentSources3D function because the path to stardist model is incorrect.
Because of this error, we could not run subsequent modules.
We would recommend the authors extend the "installation guide" to describe setting up the appropriate filepaths and editing hardcoded paths in the software (a quick fix) or develop a built-in software solution (e.g. the software could prompt the reader to update the path).

2) Another problem appears to stem from the dependence of StarDist (the default cell segmentation routine) on a pretrained model. It would improve adoption / testing of the software if a pretrained model were included in the demo data, or a walkthrough on model training. Cellpose, for example, works quite decently in our experience on many datasets using the pre-trained model without the need for further in-house training. While these in house optimization options are nice, it also greatly facilitates testing adoption / migration from other approaches, if the new approach can showcase a reasonable performance out-of-the-box.

3) Despite the advertised compatibility with cellpose as a replacement for StarDist, the installer does not appear to include this by default, nor did we find instructions for using it in place of StarDist.


Optional: we recommend the authors consider adding support for "modern" image file formats, such as OME-Zarr (HDF5 inspired methods). A substantial portion of the imaging community is trying to move beyond the limitations of TIFF (a format originally developed for FAX, whose intrinsic limitations on file size and header information limit it substantially). For further information we direct the authors to:
https://www.nature.com/articles/s41592-021-01326-w


Alistair Boettiger
&
Tee Udomlumleart

**Reviewer 2**

**Were you able to assess all statistics in the manuscript, including the appropriateness of statistical tests used?**

There are no statistics in the manuscript.

**Were you able to directly test the methods?**

No

**Comments to author:**

Manuscript GBIO-D-23-1132 describes a novel open source image analysis software named pyHiM. The goal of pyHiM is to provide end-users with an end-to-end image and data workflow for spatial genomics experiments. pyHiM includes several image analysis modules necessary for this task, including: imaging cycle registration, nucleus segmentation, spot finding and localization, and chromosomal traces construction, filtering, and analysis. In addition, the software also generates quality control and analysis reports, and includes GUI interfaces for analysis parameter setup in interactive mode, and for monitoring batch runs on high-performing computing platforms.

The manuscript is of interest to a wide group of computational biologists that work in the genome architecture field. The authors had a particular computational task at hand, and they have clearly put in a substantial amount of attention and resources to design the analysis pipeline and its interaction interfaces with the end users. The individual steps of the workflow are well explained in the main section of the text, and with meaningful examples presented as figures, which are of excellent quality. More importantly, the online documentation on how to install the software, to use the workflow, and on the usage of each of the modules and functions is extensive and detailed. Finally, I particularly appreciated the addition of downstream data analysis modules in the pipeline to produce quality control and results reports. This is an important feature that is routinely missing from most if not all open source image analysis software.

I strongly recommend the publication of manuscript GBIO-D-23-1132 in its current format in Genome Biology.

**Authors Response**

**Point-by-point responses to the reviewers’ comments:**

**Reviewer #1**

Xavier and colleagues describe a professionally organized tool for reconstructing 3D chromatin paths from sequential imaging data. Chromatin tracing data have proliferated substantially in recent years, but most practitioners still rebuild the wheel from scratch as it were, using custom in house software approaches for analysis due to the lack of easy to use reliable tools. This state of affairs has slowed the growth of the field and limited the opportunity for optimization (comparing and competing different approaches) that highly portable well documented software could bring. This work has significant promise to help close that gap and we are enthusiastic about seeing it published.

The modular nature of the work is an important strength. This is nicely showcased in the integration of different methodologies for cell-segmentation, including StarDist and cellpose. Rather than rebuild these from scratch, the software takes advantage of more widely used and well maintained existing software in the image processing field. Similar modularity is exhibited in the spot fitting with BigFISH and the use of existing generalist approaches for parallelization (with Dask) and performance monitoring (with Bokeh). The support for Windows/Mac/Linux environment was also commendable.

We are extremely thankful for the positive and constructive criticism of the manuscript, which led to a much improved version of the manuscript. Also, while unfortunately not successful, the attempts of the reviewers at testing the demo of pyHiM unveiled problems that may be encountered by many other users. By addressing these issues, we are now able to provide a more robust, and flexible release of pyHiM. We are again thankful to the reviewers for their comments and remarks.

We have the following suggestions for improvement:

While refreshingly succinct, readable, and data rich, the main text could benefit (in our opinion) from a little further development in a few key areas.

1. We found it helpful that the authors did indeed include demo data with software online. Nonetheless, the existence of such demo data was not immediately apparent from reading the main text of the article, nor from the initial readme page of the PyHiM github repository.

We thank the reviewer for catching this up. The following sentence was added to the main text, in order to further highlight the existence of the tutorials & demo data: “pyHiM comes with extensive [user and developer documentation and j](https://pyhim.readthedocs.io/en/latest/index.html)upyter notebook tutorials are also provided along with a DNA-FISH dataset to guide new users through the main steps of a typical analysis pipeline.”

Furthermore, the following sentence was added to the pyHiM repository README page: “Find the full documentation in [ReadtheDocs](https://pyhim.readthedocs.io/en/latest/) and [tutorials](https://pyhim.readthedocs.io/en/latest/getting_started/tutorials.html) illustrating the main steps of pyHiM analysis pipeline.“

2. The main text describes most of the key steps of the algorithm, but did not include a clear description of the spot-stitching approach. For example, how does the software handle the detection of multiple foci in the same image? Does it use the closest spot? The brightest spot? Some weighted combination of these?

We apologize for the lack of details on this issue. In pyHiM, inconsistencies in tracing are not handled by the main method ‘build_traces’, but rather in post-processing by dedicated scripts. For instance, *‘trace_filter’* allows for the removal of spots that appear more than once per trace, and *‘trace_filter_advanced’* allows for the selection of the spot based on distance considerations. We addressed this comment by revising the ‘Tracing’ section of the Methods and documented these scripts more thoroughly in the online documentation.

1. Completely optional: Perhaps the authors would like to add support for some recently developed (python) algorithms designed to address this issue, like the "spatial genome aligner" from the Ren lab?:<https://github.com/b2jia/jie>

We thank the reviewer for this excellent suggestion. We attempted to build a script that converts our trace table into the input format of ‘Spatial genome aligner’. Unfortunately, the ‘Spatial genome aligner’ does not seem to provide a standard input format for chromatin traces. Neither the paper, nor the online repository describe the existence of such a format, and the online tutorials perform explicit format conversions for different multiplexed DNA-FISH datasets. Our chromatin trace format contains all the information necessary to reformat it into the variables used by ‘Spatial genome aligner’, thus, we just provide the following statement in the text: “We highlight the existence of more sophisticated methods to improve the quality of tracing [^24^](https://paperpile.com/c/jloBXk/0Zpl) that may be applied in combination with the post-processing scripts mentioned in this paragraph.”

2. Despite the elegant implementation with conda and pip, and the reasonable amount of documentation, we were ultimately unable to run even the demo data analysis "out-of-the-box". These errors no doubt reflect our own deficiencies as much as they reflect any inadequacies in the author's approach. Nonetheless, in hopes of helping improve the software and its adaptation, we summarize the problems we encountered here:

One problem appears to stem from some filepaths that are still hard coded:

*Cannot run segmentMask3d function FileNotFoundError: config file doesn't exist: /mnt/grey/DATA/users/marcnol/models/StarDist3D/mask_DAPI/models/stardist_20210625_deconvolved/config.js*

We apologize for this problem, which arose because the paths of the trained networks in the local system were not indicated in the parameters.json file. Therefore pyHiM tried to use default values which pointed to our own filesystem. This clearly reflected inadequacies in our documentation.

To remove this problem altogether, we now include the trained networks as part of the package. Therefore, in the revised version of pyHiM there is no longer a need for the user to download our trained networks to their local file system. Still, the user can provide their own trained networks by indicating their location within the input parameters.json file.

3. Similarly, I cannot run segmentSources3D function because the path to stardist model is incorrect. Because of this error, we could not run subsequent modules.

We would recommend the authors extend the "installation guide" to describe setting up the appropriate filepaths and editing hardcoded paths in the software (a quick fix) or develop a built-in software solution (e.g. the software could prompt the reader to update the path).

This is part of the same issue raised in Remark #4. We imagine trained networks were not downloaded and their path was not indicated in parameters.json. We apologize for the poor documentation leading to this issue.

In the new release of pyHiM, trained networks are included within the package, therefore this issue will no longer exist. As indicated in the answer above, the user can still provide their own networks in the parameters.json input file, and pyHiM will raise an error if these user-trained networks cannot be found.

4. Another problem appears to stem from the dependence of StarDist (the default cell segmentation routine) on a pretrained model. It would improve adoption / testing of the software if a pretrained model were included in the demo data, or a walkthrough on model training. Cellpose, for example, works quite decently in our experience on many datasets using the pre-trained model without the need for further in-house training. While these in house optimization options are nice, it also greatly facilitates testing adoption / migration from other approaches, if the new approach can showcase a reasonable performance out-of-the-box.

In the new release of pyHiM, networks are included as part of the installation package, therefore the execution of the demo data will no longer require the user to download these networks.

In addition, we now provide and document a script to use cellpose to segment nuclear masks (script: [mask_cellpose.py](https://pyhim.readthedocs.io/en/latest/user_guide/modules/identification/mask_cellpose.html), [tutorial](https://pyhim.readthedocs.io/en/latest/getting_started/tutorials/cellpose.html)). This modular approach opens the possibility to segment images using other AI tools that may arise in future and that can be similarly incorporated within the pyHiM pipeline.

5. Despite the advertised compatibility with cellpose as a replacement for StarDist, the installer does not appear to include this by default, nor did we find instructions for using it in place of StarDist.

We apologize for the lack of clarity of how to use cellpose in the original submission. To clarify this, we wrote a stand-alone script (mask_cellpose.py) that loads and segments any image file using cellpose, using user-provided parameters (e.g. model type, cell_prob, flow_threshold, stitch_threshold, or diameter). The documentation of this script can be found at: [mask_cellpose.py](https://pyhim.readthedocs.io/en/latest/user_guide/modules/identification/mask_cellpose.html). In addition, we now provide a tutorial to show new users how to use this script: <https://pyhim.readthedocs.io/en/latest/getting_started/tutorials/cellpose.html>.

6. Optional: we recommend the authors consider adding support for "modern" image file formats, such as OME-Zarr (HDF5 inspired methods). A substantial portion of the imaging community is trying to move beyond the limitations of TIFF (a format originally developed for FAX, whose intrinsic limitations on file size and header information limit it substantially). For further information we direct the authors to:

<https://www.nature.com/articles/s41592-021-01326-w>

We agree with the reviewer in that this would be a very interesting support to add to pyHiM. We have investigated which of the available formats would be more suitable, and realized that many similar ‘modern’ formats are currently being used by the community (ZARR, OME-ZARR, HDF5) therefore we think the best would be to implement support for all these formats in an upcoming release.

**Reviewer #2**

Manuscript GBIO-D-23-1132 describes a novel open source image analysis software named pyHiM. The goal of pyHiM is to provide end-users with an end-to-end image and data workflow for spatial genomics experiments. pyHiM includes several image analysis modules necessary for this task, including: imaging cycle registration, nucleus segmentation, spot finding and localization, and chromosomal traces construction, filtering, and analysis. In addition, the software also generates quality control and analysis reports, and includes GUI interfaces for analysis parameter setup in interactive mode, and for monitoring batch runs on high-performing computing platforms.

The manuscript is of interest to a wide group of computational biologists that work in the genome architecture field. The authors had a particular computational task at hand, and they have clearly put in a substantial amount of attention and resources to design the analysis pipeline and its interaction interfaces with the end users. The individual steps of the workflow are well explained in the main section of the text, and with meaningful examples presented as figures, which are of excellent quality. More importantly, the online documentation on how to install the software, to use the workflow, and on the usage of each of the modules and functions is extensive and detailed. Finally, I particularly appreciated the addition of downstream data analysis modules in the pipeline to produce quality control and results reports. This is an important feature that is routinely missing from most if not all open source image analysis software.

I strongly recommend the publication of manuscript GBIO-D-23-1132 in its current format in Genome Biology.

We thank the reviewer for this very positive review of our submission.

**Second round of review**

**Reviewer 1**

The authors have carefully addressed all our concerns.
